# Parallel evolution of chimeric genes in microbial evolution experiments

**DOI:** 10.1099/mgen.0.001850

**Published:** 2026-09-30

**Authors:** Rohan Maddamsetti, Mehmet Orman

**Affiliations:** 1 Department of Biochemistry and Microbiology, Rutgers University, New Brunswick, NJ, USA; 2 Department of Biomedical Engineering, University of Wisconsin, Madison, WI, USA

**Keywords:** antibiotic resistance, chimeric genes, experimental evolution, genomic deletions, prophage, structural variation

## Abstract

Recombination can generate new or improved proteins by merging pieces of pre-existing genes into a new whole. Here, when we reanalysed genomic data from several laboratory evolution experiments with *Escherichia coli*, evidence for the convergent evolution of chimeric genes became apparent. In these experiments, a pair of paralogous genes was recombined into a single hybrid copy by large genomic deletions. In one case, the excision of a cryptic e14 prophage occurred in 6 out of 8 replicates of a 22-day fluoroquinolone-resistance evolution experiment. These parallel prophage excisions recombined an *icd* isocitrate dehydrogenase gene with a homologous *icdC* C-terminal fragment pseudogene. In the other case, convergent 23 kbp deletions recombined *cpsG* paralogs across replicate populations of the Lenski long-term evolution experiment with *E. coli*, generating a chimeric phosphomannomutase. Together, these results indicate that chimeric genes evolve rapidly in bacteria and are more common than previously believed because they are easily missed by standard genomic analyses. Experiments are needed to determine whether these chimeric proteins are adaptive or are merely by-products of their associated genomic deletions.

## Data Summary

All data used in this study are publicly available. All data, code and results for this study are available on Zenodo (DOI: 10.5281/zenodo.20692138). Genomic data published by Mohiuddin *et al*. are available in the European Nucleotide Archive (Study ERP166963 with Study Accession PRJEB83321), and genomic data published by Tenaillon *et al*. are available from the NCBI BioProject database under accession number PRJNA294072.

**Impact Statement**

Genomic deletions can generate chimeric proteins by splicing together parts of existing genes. However, the extent to which chimeric proteins form and the extent to which they are adaptive, are not well understood. Complex structural variants (SVs) (such as chimeric protein formation) are often missed or incompletely annotated by standard genomic pipelines. Furthermore, there is an ongoing need for new methods to detect selection on complex SVs. In this work, we report evidence of chimeric gene formation in laboratory evolution experiments with *Escherichia coli*. In both experiments, chimeric genes evolved in parallel across independent replicate populations, which is an unambiguous signal of positive selection (although it remains unclear whether selection is operating on the chimeric gene itself, or on linked genomic mutations). The observation of chimeric gene formation in laboratory experiments indicates that chimeric genes evolve rapidly in bacteria and are more common than previously believed. Therefore, this work points to an important new direction for microbial genomics research: what is the extent of chimeric gene formation in natural bacterial populations and what consequences might chimeric genes have for bacterial adaptation?

## Introduction

New genes and functions often evolve by duplicating, inserting, deleting and recombining parts of existing genes, leading to functional innovation [1–3]. The potential for recombination to generate new genes has long been appreciated in eukaryotes [4, 5]. By contrast, the extent to which duplications, deletions and recombination generate adaptive protein variation in bacteria is less understood, for several reasons. First, many forms of copy number variation in bacteria (such as tandem gene amplifications) [6, 7] are highly unstable and compete with horizontal gene transfer as a means for gaining new functions [8, 9]. Second, some mechanisms for generating diverse alleles in bacterial populations are well understood but remain poorly quantified. For instance, it is well known that intragenomic recombination between duplicated genes (gene conversion) generates protein diversity and antigenic variation in many pathogenic bacteria [10–15]. The extent of gene conversion dynamics across the bacterial tree of life remains unknown, however, because short-read sequencing is often unable to resolve the genomic location of repeat sequences [16]. Thanks to ongoing advances in genome sequencing technology [17], it has become clear that recombination and structural variation are pervasive in natural bacterial populations [18] and during microbiome evolution [19], and indeed, that structural variation is pervasive across the tree of life [20]. For example, two recent studies used long-read sequencing to discover that gene conversion and duplication are key drivers of diversity hotspots in antigens and virulence-associated loci in *Mycobacterium tuberculosis*, an organism in which recombination was previously thought to play a minimal role [21, 22]. Finally, the measurement of some mechanisms of gene formation often requires examining existing data in new ways [23]. For example, genomic deletions are usually considered as a mechanism that streamlines genomes by removing unneeded genes. However, interstitial genomic deletions can in fact yield new genes. There are two primary mechanisms. First, a deletion can yoke together parts of unrelated genes, forming a new protein by gene fusion [24, 25]. Second, a deletion can yoke together different parts of homologous genes (such as paralogs formed by recent gene duplications), forming a new chimeric gene.

In this paper, we describe two cases of chimeric gene formation by this second mechanism across different evolution experiments with *Escherichia coli*. The chimeric alleles that evolved in these evolution experiments have also been found among natural *E. coli* isolates, due to recurrent recombination, HGT and deletion events at these loci [26–34]. These findings suggest chimeric gene formation may be more common than previously believed because many more cases of chimeric gene formation in bacteria may have gone undetected in existing genomic studies of bacterial evolution in the laboratory and in nature.

## Results

### Parallel evolution of a chimeric isocitrate dehydrogenase during experimental evolution under fluoroquinolone exposure and recovery

Mohiuddin *et al*. carried out an evolution experiment with *E. coli* K-12 to investigate adaptation to fluoroquinolone stress. In this 22-day experiment, eight replicate populations were subjected to multiple cycles of high-dose ofloxacin exposure followed by drug-free recovery [35]. Whole-genome sequencing of each evolved population revealed limited evidence of convergent evolution. This result was surprising, because parallel evolution is a more typical outcome for short-term evolution experiments with large population sizes and strong selection pressures, such as antibiotic use [36]. Since parallel evolution is mediated by mutations with either large fitness benefits or high rates [37, 38], we asked whether some kinds of mutations (such as gene copy number changes) may have been missed by the initial analysis, which focused on SNPs, insertions and deletions (indels) and structural variants.

To answer this question, we re-analysed these genomic data using *breseq* [39–41] and additional statistical methods to detect copy number variation [36, 42]. While we did find evidence of parallel 2–3× gene copy number amplifications of the *bamA*, *rpoB* and *lldP* genes (Table S1, available in the online Supplementary Material), we further examined SNPs in the *icd* isocitrate dehydrogenase gene because *icd* has been implicated in persistence and antibiotic tolerance [43] and has been reported to be recurrently mutated in genomic studies of tolerant populations [44]. Six out of the eight evolved populations show multiple identical synonymous changes to *icd*, including mutations affecting the same codon. In addition, these synonymous changes correlated perfectly with missing sequencing coverage affecting the C-terminus of *icd*, indicating parallel 15.1 kbp deletions (Table 1). By examining the K-12 MG1655 genome in the Benchling [45] and Proksee [46] genome browsers, we found that these putative deletions correlated perfectly with the coordinates of cryptic prophage e14 in the MG1655 genome (Table 1). Notably, e14 is a known prophage element in *E. coli* K-12 that has been shown to excise under induction of the SOS response [47–49] and has been linked to *icd* mutations in ciprofloxacin-evolved populations [50]. Further visual inspection of the *icd* and prophage e14 locus in MG1655 revealed that cryptic prophage e14 is flanked by *icd* at one end, which extends into the e14 prophage and by an *icdC* C-terminal pseudogene at the other end of the e14 prophage, consistent with observations reported by Hill *et al*. [49] and Sulaiman *et al*. [50] (Fig. 1a). To further test if the parallel 15.1 kbp deletions of prophage e14 can recombine *icd* with *icdC*, we modelled the effect of deleting the region spanning 1196266–1211412 in the MG1655 genome on the *icd* gene and aligned the resulting chimeric protein against its parental genes *icd* and *icdC* (Fig. 1b). The resulting protein has a duplicated MLRHMGWTEAADLIVKGME C-terminal sequence and D417E and D429E substitutions through recombination with *icdC*, but is otherwise identical to the translated protein product of *icd* (Fig. 1b).

**Fig. 1.**
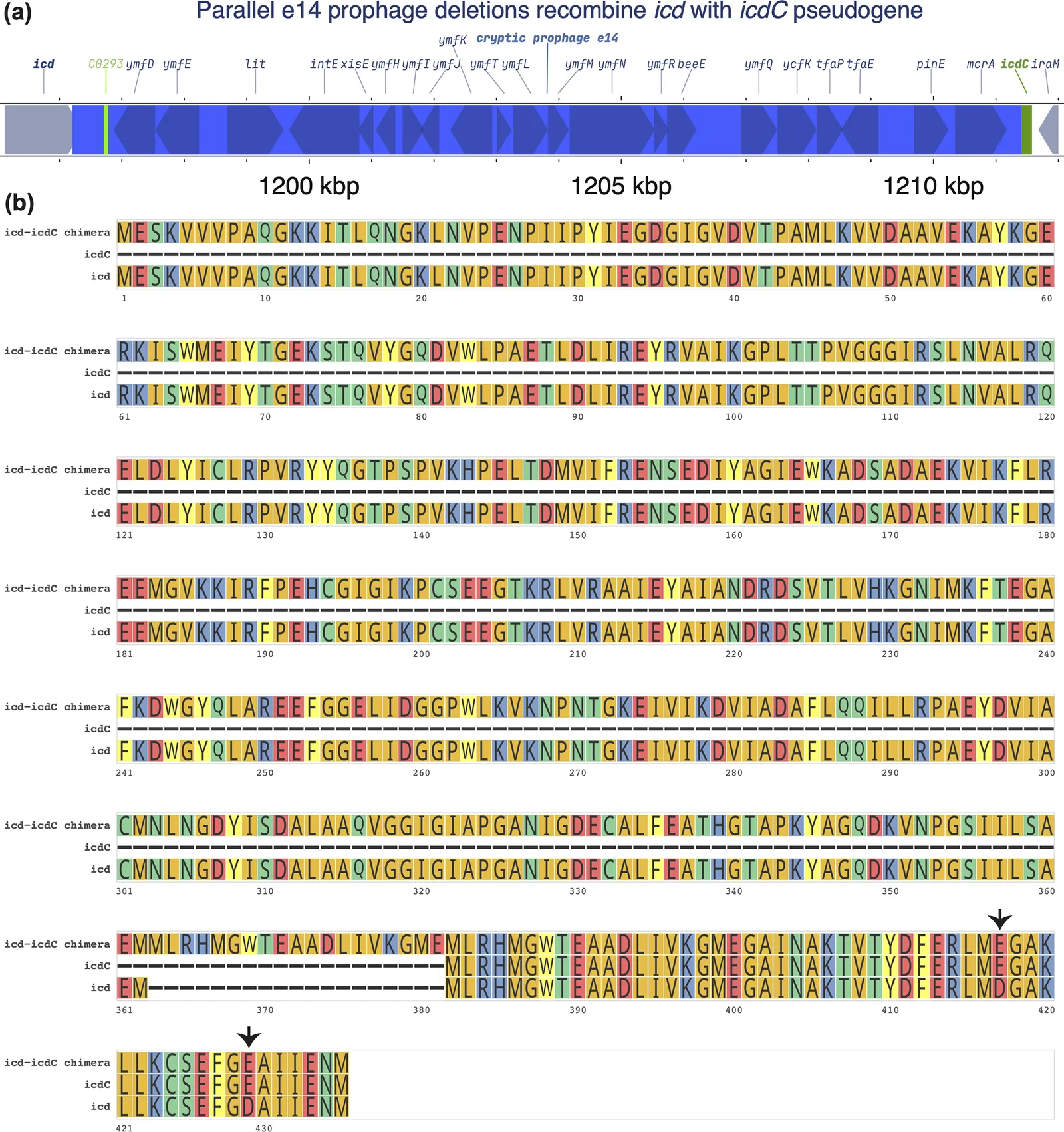
Parallel e14 prophage deletions recombine *icd* with *icdC* pseudogene during experimental evolution of fluoroquinolone resistance. (**a**) Structure of pre-deletion locus in the *E. coli* K-12 MG1655 ancestral clone. Protein-coding genes are coloured grey-blue, a small RNA is coloured light green, the *icdC* pseudogene is coloured dark green and e14 prophage is coloured blue. (**b**) Multiple protein sequence alignment of chimeric *icd-icdC* with its parental *icd* and *icdC* genes. Arrows indicate *icdC* aa substitutions introduced by recombination.

**Table 1.** Parallel synonymous changes in *icd* isocitrate dehydrogenase correlate with loss of cryptic prophage e14 (1196209–1211412) during experimental evolution of fluoroquinolone resistance [35]

Population	Position	*icd* Haplotype	Missing coverage start	Missing coverage end	Deletion size (bp)
1	1196220	H366H (CAC→CAT)	1196266	1211412	15147
	1196232	T370T (ACC→ACT)			
2	1196220	H366H (CAC→CAT)	1196266	1211412	15147
	1196232	T370T (ACC→ACT)			
	1196245	L375L (TTA→CTG)			
	1196247	L375L (TTA→CTG)			
3	1196220	H366H (CAC→CAT)	1196260	1211417	15158
	1196232	T370T (ACC→ACT)			
	1196245	L375L (TTA→CTG)			
	1196247	L375L (TTA→CTG)			
5	1196220	H366H (CAC→CAT)	1196268	1211412	15145
	1196232	T370T (ACC→ACT)			
	1196245	L375L (TTA→CTG)			
	1196247	L375L (TTA→CTG)			
7	1196220	H366H (CAC→CAT)	1196266	1211430	15165
	1196232	T370T (ACC→ACT)			
	1196245	L375L (TTA→CTG)			
	1196247	L375L (TTA→CTG)			
8	1196220	H366H (CAC→CAT)	1196271	1211426	15156
	1196232	T370T (ACC→ACT)			
	1196245	L375L (TTA→CTG)			
	1196247	L375L (TTA→CTG)			

### Parallel losses of the rfb O-antigen (O-polysaccharide) biosynthesis gene cluster drive evolution of chimeric phosphomannomutase in the long-term evolution experiment

A similar phenomenon occurs in the Lenski long-term evolution experiment with *E. coli* (LTEE). Parallel 23 kbp genomic deletions occurred in 8 of the 12 replicate populations of the LTEE [51] (Table 2). These 23 kbp deletions recombine two highly similar phosphomannomutases involved in GDP-mannose synthesis: *cpsG*, which is part of a biosynthetic gene cluster making the extracellular polysaccharide colanic acid [52], and *manB,* which is part of the O-antigen gene cluster [27, 28]. Visual inspection of this region of the *E. coli* B str. REL606 genome in the Benchling [45] and Proksee [46] genome browsers shows that these deletions affect the *rfb* O-antigen (O-polysaccharide) biosynthesis gene cluster, although this strain already does not produce O-specific side-chain lipopolysaccharide [53] (Fig. 2a). We examined the effect of this Δ23,293 bp mutation on *cpsG* starting from position 2,031,703, as this variant occurs in two LTEE populations, Ara+4 and Ara−1 (Table 2). An alignment of the resulting chimeric protein shows that the deletion recombines the N-terminus of CpsG with the C-terminus of ManB (Fig. 2b).

**Fig. 2.**
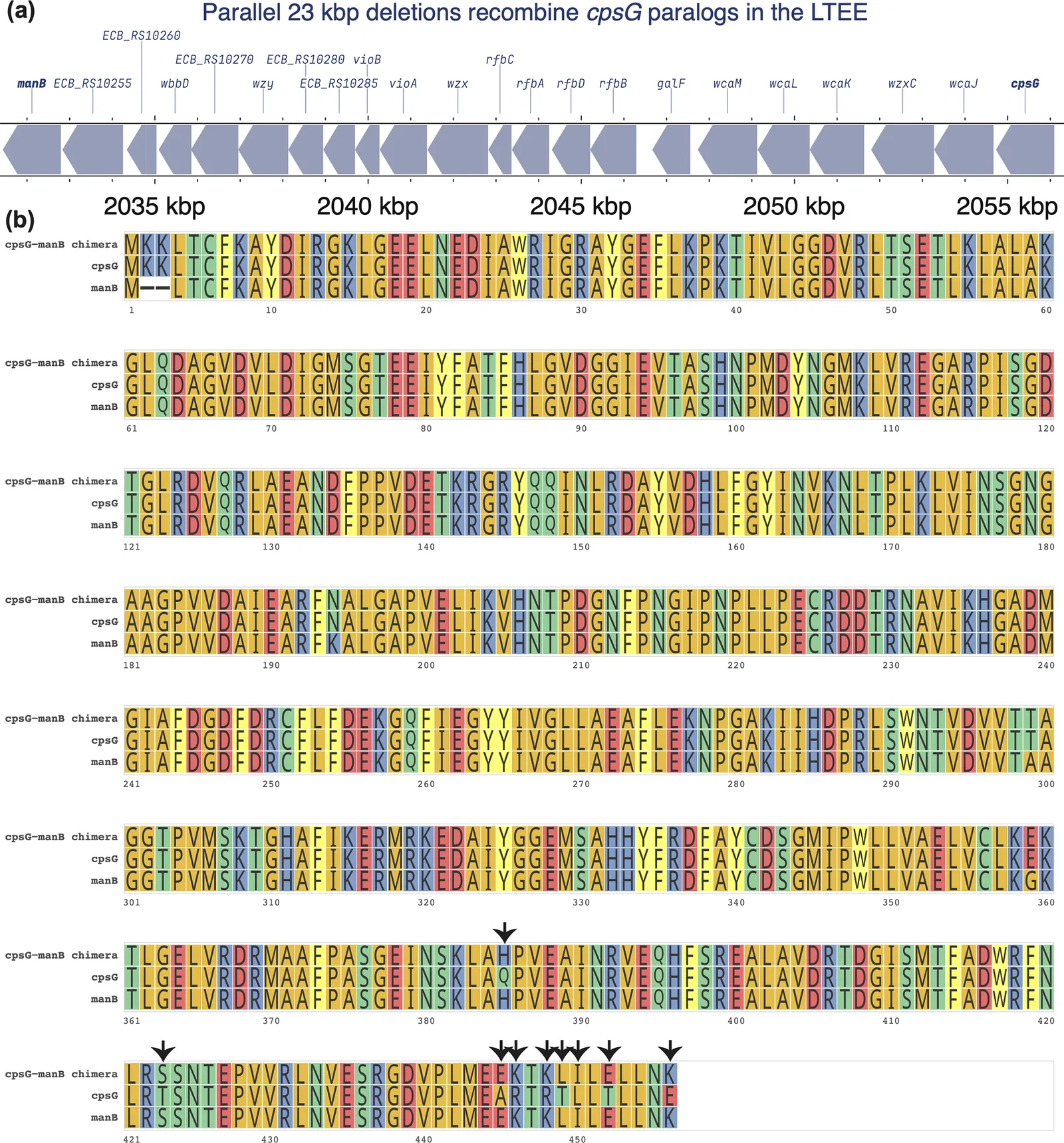
Parallel 23 kbp deletions recombine *cpsG* paralogs in the LTEE. (**a**) Structure of pre-deletion locus in the genome of the REL606 LTEE ancestral clone. Protein-coding genes are coloured grey-blue. (b) Multiple sequence alignment of the chimeric *cpsG-manB* phosphomannomutase with its parental *cpsG* and *manB* genes. Arrows indicate *manB* aa substitutions introduced by recombination.

**Table 2.** Parallel genomic deletions between *manB* and *cpsG* phosphomannomutase paralogs in the LTEE*

Population	Generation first observed	Fixation	Position	Mutation (bp)	Gene
Ara+3	10,000	Yes	2,032,210	Δ23,293	*[manB]–[cpsG]*
Ara+4	15,000	Yes	2,031,703	Δ23,293	*[manB]–[cpsG]*
Ara−1	10,000	Yes	2,031,703	Δ23,293	*[manB]–[cpsG]*
Ara+5	20,000	No	2,032,369	Δ23,293	*[manB]–[cpsG]*
Ara−2 (L†)	5000	Yes	2,032,399	Δ23,293	*[manB]–[cpsG]*
Ara−2 (S†)	15,000	Yes	2,031,537	Δ23,293	*[manB]–[cpsG]*
Ara−3	20,000	Yes	2,032,711	Δ23,293	*[manB]–[cpsG]*
Ara−4	10,000	Yes	2,032,114	Δ23,293	*[manB]–[cpsG]*
Ara−5	15,000	Yes	2,032,762	Δ23,293	*[manB]–[cpsG]*

* Data about ‘large’ and ‘small’ colony-forming ecotypes in the Ara−2 population retrieved from Supplementary Data 1 of Tenaillon *et al*. (2016).

† Data retrieved from https://barricklab.org/shiny/LTEE-Ecoli/ on 4 May 2026.

## Discussion

Here, we describe the parallel evolution of chimeric proteins via large genomic deletions in laboratory evolution experiments. In both cases, the large deletion was flanked by two highly homologous gene sequences, likely derived by recent gene duplication. Recombination between those paralogs joined the N-terminus of one protein with the C-terminus of the other, removing all intervening genes (Fig. 3).

**Fig. 3.**
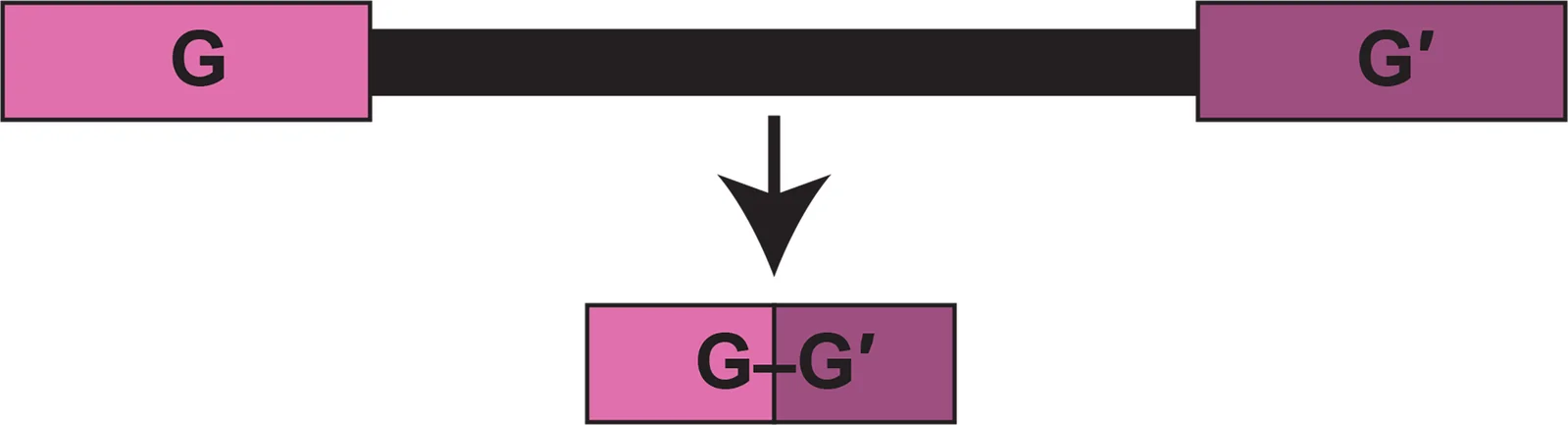
Large genomic deletions recombine paralogous genes G and G′, resulting in chimeric gene G–G′.

Some aspects of these events were reported previously, such as the occurrence of parallel SNPs in the evolution experiments by Mohiuddin *et al*. [35] and Sulaiman and Lam [50] and the occurrence of parallel genomic deletions in the LTEE [51]. However, the evolution of chimeric isocitrate dehydrogenase was not explicitly recognized by Mohiuddin *et al*. because two separate observations had to be combined to make this inference: an anomalous pattern of parallel synonymous substitutions, coupled with loss of sequencing coverage at a neighbouring cryptic e14 prophage [35]. In addition, while Sulaiman and Lam [50] recognized the role of the e14 prophage in causing parallel SNPs in *icd*, they erroneously inferred that the cause of these SNPs was e14 prophage *integration*, rather than e14 *excision* from the *E. coli* K-12 MG1655 genome. In the second case, it was known that the parallel 23 kbp deletions affected *cpsG* homologs in the LTEE, but the resulting chimeric *cpsG* gene sequences had not been studied further or reported in any publications [51].

The chimeric alleles that evolved in these evolution experiments have been found among many natural *E. coli* isolates, due to recurrent recombination, HGT and deletions at both *icd* and the genomic region containing *manB* and *cpsG* [26–34]. Still, we speculate that many more cases of chimeric gene evolution may have occurred but gone undetected in previous experimental evolution studies. Gold-standard microbial genome analysis pipelines like *breseq* report deletions as well as synonymous mutations introduced by recombination, but to our knowledge, no current genomic pipelines directly annotate new chimeric proteins that arise in evolution experiments. Furthermore, variation at recently duplicated genes remains largely uncharacterized in evolution experiments. For instance, in the context of the LTEE, the dynamics of genetic variation at *manB* and *cpsG* are poorly understood, because large-scale genomic and metagenomic studies of the LTEE filtered out all genetic variation in repetitive regions, including multiple direct sequence repeats shared by *manB* and *cpsG* [51, 54].

This work leaves open the question of whether the parallel evolution was driven by positive selection on the chimeric gene, positive selection on the deletion or positive selection on unknown genomic variants linked to the deletion and associated chimeric gene. The first two cases are straightforward to understand. The third case would happen if the deletion and associated chimeric gene have no selective benefit but recur at a sufficiently high rate that they eventually hitchhike to fixation across replicate experimental populations [55–58]. Of these three hypotheses, the second explanation (selection on the deletion) is most parsimonious. Large genomic deletions can be beneficial because they remove unneeded proteins. They can also occur at high rates due to homology between direct genetic repeats, as found in paralogous gene copies. Genomic deletions could also occur at high rates due to the induction of site-specific recombination systems, such as those present in some prophages [59]. This seems to be the case for e14 prophage excision and *icd* chimera formation, which are specifically induced by the SOS DNA damage response [47–49, 59]. Nevertheless, the weight of current evidence suggests that positive selection is still a key driver for the parallel evolution of *icd* chimera formation in the experiments conducted by Mohiuddin *et al*. [35] and by Sulaiman *et al*. [50] because e14 is excised slowly and/or inefficiently after SOS induction [47, 48]. Further experiments with isogenic *E. coli* K-12 strains with and without excised e14 prophage are needed to test this hypothesis and directly measure the effects of chimeric *icd* on bacterial fitness, antibiotic tolerance and persister phenotype formation [35, 50].

The conservative genetic changes observed in both protein alignments (Figs 1b and 2b) suggest that both chimeric proteins would be functional if they are indeed expressed. Supporting this interpretation, Hill *et al*. reported the results of isocitrate dehydrogenase assays on cell lysates isolated before and after e14 excision by SOS induction [49]. Their report indicates that the chimeric *icd* after e14 excision is both expressed and functional, albeit with marginally lower activity than ancestral *icd* before e14 excision [49]. More generally, it is certainly possible that chimeric proteins formed by large genomic deletions could be highly beneficial, given the analogous finding that recombination between infectious lytic phage and a defunct prophage can dramatically improve phage fitness during coevolutionary phage training [60].

Recent work by Kaul *et al*. has reported the evolution of novel gene fusions by large genomic deletions in both the LTEE and across the microbial tree of life [24]. In addition, Uz-Zaman *et al*. have reported evidence of *de novo* gene birth in the LTEE [61]. This paper reports an additional mechanism that may contribute to novel genetic variation in microbial evolution experiments and points to the need for quantitative estimates of the prevalence and importance of each mechanism for the evolution of novel genetic functions across the tree of life.

## Methods

### Genomic analysis of a 22-day fluoroquinolone-resistance evolution experiment

Genomic data published by Mohiuddin *et al*. [35] were downloaded from ArrayExpress (https://www.ebi.ac.uk/biostudies/arrayexpress/studies/E-MTAB-14682, last accessed 13 May, 2026). Also see Study ERP166963 (https://www.ebi.ac.uk/ena/browser/view/ERP166963, last accessed 13 May, 2026) with Study Accession PRJEB83321 in the European Nucleotide Archive. *breseq* v0.37 was used to call mutations in these genomes using the *E. coli* K-12 MG1655 reference genome in NCBI RefSeq (GCF_000005845.2_ASM584v2). Mutations in the resequenced ancestral K-12 clone used for this experiment were applied to the NCBI RefSeq genome using the *gdtools* utility programme distributed with *breseq* to generate an updated reference genome, which was then used to analyse mutations in each endpoint population. Resulting genomediff-format output files produced by *breseq* were collated using a Python 3.12 script called *process-gdiffs.py* and statistical evidence for gene copy number amplifications was analysed using an R 4.5.2 script called *copy-number-analysis.R*. Ancestral and evolved *icd* and *icdC* sequences were extracted from the K-12 reference genome and aligned using MAFFT [62] v7.526 with a Python 3.12 script called *extract_and_align_icd_seqs.py*.

### Genomic analysis of manB-cpsG deletions in the LTEE

Mutations in evolved LTEE genomes were retrieved from https://barricklab.org/shiny/LTEE-Ecoli/ on 4 May 2026. Ancestral and evolved *cpsG* homologs were extracted from the *E. coli* B str. REL606 reference genome and aligned using MAFFT [62] v7.526 with a Python 3.12 script called *extract_and_align_cpsG_seqs.py*.

### Data visualization

The ancestral loci in the K-12 and REL606 genomes before chimeric gene formation by interstitial deletions were visualized using Proksee [46] and Benchling [45]. Visualizations of protein alignments were made using R 4.5.2 scripts called *plot_icd_alignment.R* and *plot_manB-cpsG_alignment.R*.

## Supplementary material

10.1099/mgen.0.001850 Supplementary Material 1.

## Abbreviations

aa: amino acid
bp: base pair
HGT: horizontal gene transfer
indel: insertion/deletion
kbp: kilobase pair
LTEE: long-term evolution experiment
SNP: single-nucleotide polymorphism
SV: structural variant

## References

[1] Conant GC, Wagner A (2005). The rarity of gene shuffling in conserved genes. Genome Biol.

[2] Xia S, Chen J, Arsala D, Emerson JJ, Long M (2025). Functional innovation through new genes as a general evolutionary process. Nat Genet.

[3] Silhavy TJ (2000). Gene Fusions. J Bacteriol.

[4] Rogers RL, Bedford T, Hartl DL (2009). Formation and longevity of chimeric and duplicate genes in *Drosophila melanogaster*. Genetics.

[5] Smithers B, Oates M, Gough J (2019). ‘Why genes in pieces?’—revisited. Nucleic Acids Res.

[6] Andersson DI, Hughes D (2009). Gene amplification and adaptive evolution in bacteria. Annu Rev Genet.

[7] Jonsson S, Guliaev A, Berryhill BA, Andersson DI, Nicoloff H (2026). The dynamic distribution of genetic tandem amplifications in a heteroresistant *Escherichia coli* population revealed by ultra-deep long read sequencing. Nat Commun.

[8] Treangen TJ, Abraham A-L, Touchon M, Rocha EPC (2009). Genesis, effects and fates of repeats in prokaryotic genomes. FEMS Microbiol Rev.

[9] Treangen TJ, Rocha EPC (2011). Horizontal transfer, not duplication, drives the expansion of protein families in prokaryotes. PLoS Genet.

[10] Santoyo G, Romero D (2005). Gene conversion and concerted evolution in bacterial genomes. FEMS Microbiol Rev.

[11] Palmer GH, Brayton KA (2007). Gene conversion is a convergent strategy for pathogen antigenic variation. Trends Parasitol.

[12] Graves CJ, Ros VID, Stevenson B, Sniegowski PD, Brisson D (2013). Natural selection promotes antigenic evolvability. PLoS Pathog.

[13] Bergholz TM, Tarr CL, Christensen LM, Betting DJ, Whittam TS (2007). Recent gene conversions between duplicated glutamate decarboxylase genes (gadA and gadB) in pathogenic *Escherichia coli*. Mol Biol Evol.

[14] Nystedt B, Frank AC, Thollesson M, Andersson SGE (2008). Diversifying selection and concerted evolution of a type IV secretion system in *Bartonella*. Mol Biol Evol.

[15] Tsuru T, Kobayashi I (2008). Multiple genome comparison within a bacterial species reveals a unit of evolution spanning two adjacent genes in a tandem paralog cluster. Mol Biol Evol.

[16] Moss EL, Maghini DG, Bhatt AS (2020). Complete, closed bacterial genomes from microbiomes using nanopore sequencing. Nat Biotechnol.

[17] Ahsan MU, Liu Q, Perdomo JE, Fang L, Wang K (2023). A survey of algorithms for the detection of genomic structural variants from long-read sequencing data. Nat Methods.

[18] Preska Steinberg A, Kussell E (2025). How recombination and clonal evolution shape bacterial lineages and genomes. Genetics.

[19] Wolff R, Garud NR (2026). Gene-specific selective sweeps are pervasive across human gut microbiomes. Nature.

[20] Chen L, Zhao N, Cao J, Liu X, Xu J (2022). Short- and long-read metagenomics expand individualized structural variations in gut microbiomes. Nat Commun.

[21] Stritt C, Reitsma M, Marin AMG, Goig G, Dötsch A (2025). Gene conversion and duplication contribute to genetic variation in an outbreak of *Mycobacterium tuberculosis*. Microb Genom.

[22] Marin MG, Quinones-Olvera N, Jin H, Harris MA, Jeffrey BM (2026). Gene conversion is a key driver of diversity hotspots in *M. tuberculosis* antigens and virulence-associated loci. bioRxiv.

[23] Watson AK, Lopez P, Bapteste E (2022). Hundreds of out-of-frame remodeled gene families in the *Escherichia coli* pangenome. Mol Biol Evol.

[24] Kaul A, Rossine F, Břinda K, Baym M (2026). Novel genes arise from genomic deletions across the bacterial tree of life. bioRxiv.

[25] Panagopoulos I, Heim S (2021). Interstitial deletions generating fusion genes. Cancer Genomics Proteomics.

[26] Campbell A, Schneider SJ, Song B (1992). Lambdoid phages as elements of bacterial genomes (Integrase/phage21/*Escherichia coli* K-12/icd gene). Genetica.

[27] Jensen SO, Reeves PR (2004). Deletion of the *Escherichia coli* O14:K7 O antigen gene cluster. Can J Microbiol.

[28] Jensen SO, Reeves PR (2001). Molecular evolution of the GDP-mannose pathway genes (manB and manC) in *Salmonella enterica*. Microbiology.

[29] Wang F-S, Whittam TS, Selander RK (1997). Evolutionary genetics of the isocitrate dehydrogenase gene (ICD) in *Escherichia coli* and *Salmonella enterica*. J Bacteriol.

[30] Milkman R, Jaeger E, McBride RD (2003). Molecular evolution of the *Escherichia coli* chromosome. VI. Two regions of high effective recombination. Genetics.

[31] Touchon M, Hoede C, Tenaillon O, Barbe V, Baeriswyl S (2009). Organised genome dynamics in the *Escherichia coli* species results in highly diverse adaptive paths. PLoS Genet.

[32] Yoon SH, Han M-J, Jeong H, Lee CH, Xia X-X (2012). Comparative multi-omics systems analysis of *Escherichia coli* strains B and K-12. Genome Biol.

[33] Ingle DJ, Valcanis M, Kuzevski A, Tauschek M, Inouye M (2016). In silico serotyping of *E. coli* from short read data identifies limited novel O-loci but extensive diversity of O:H serotype combinations within and between pathogenic lineages. Microb Genom.

[34] Didelot X, Méric G, Falush D, Darling AE (2012). Impact of homologous and non-homologous recombination in the genomic evolution of *Escherichia coli*. BMC Genomics.

[35] Mohiuddin SG, Kavousi P, Figueroa D, Ghosh S, Orman MA (2025). The diverse phenotypic and mutational landscape induced by fluoroquinolone treatment. mSystems.

[36] Maddamsetti R, Yao Y, Wang T, Gao J, Huang VT (2024). Duplicated antibiotic resistance genes reveal ongoing selection and horizontal gene transfer in bacteria. Nat Commun.

[37] Levy SF, Blundell JR, Venkataram S, Petrov DA, Fisher DS (2015). Quantitative evolutionary dynamics using high-resolution lineage tracking. Nature.

[38] Schenk MF, Zwart MP, Hwang S, Ruelens P, Severing E (2022). Population size mediates the contribution of high-rate and large-benefit mutations to parallel evolution. *Nat Ecol Evol*.

[39] Deatherage DE, Barrick JE (2014). Engineering and Analyzing Multicellular Systems: Methods and Protocols 165–188.

[40] Barrick JE, Colburn G, Deatherage DE, Traverse CC, Strand MD (2014). Identifying structural variation in haploid microbial genomes from short-read resequencing data using breseq. BMC Genomics.

[41] Deatherage DE, Traverse CC, Wolf LN, Barrick JE (2014). Detecting rare structural variation in evolving microbial populations from new sequence junctions using breseq. Front Genet.

[42] Blount ZD, Maddamsetti R, Grant NA, Ahmed ST, Jagdish T (2020). Genomic and phenotypic evolution of *Escherichia coli* in a novel citrate-only resource environment. Elife.

[43] Manuse S, Shan Y, Canas-Duarte SJ, Bakshi S, Sun W-S (2021). Bacterial persisters are a stochastically formed subpopulation of low-energy cells. PLoS Biol.

[44] Lopatkin AJ, Bening SC, Manson AL, Stokes JM, Kohanski MA (2021). Clinically relevant mutations in core metabolic genes confer antibiotic resistance. Science.

[45] (2026). Benchling [Biology Software]. https://benchling.com.

[46] Grant JR, Enns E, Marinier E, Mandal A, Herman EK (2023). Proksee: in-depth characterization and visualization of bacterial genomes. Nucleic Acids Res.

[47] Greener A, Hill CW (1980). Identification of a novel genetic element in *Escherichia coli* K-12. J Bacteriol.

[48] Brody H, Hill CW (1988). Attachment site of the genetic element e14. J Bacteriol.

[49] Hill CW, Gray JA, Brody H (1989). Use of the isocitrate dehydrogenase structural gene for attachment of e14 in *Escherichia coli* K-12. J Bacteriol.

[50] Sulaiman JE, Lam H (2020). Proteomic investigation of tolerant *Escherichia coli* populations from cyclic antibiotic treatment. J Proteome Res.

[51] Tenaillon O, Barrick JE, Ribeck N, Deatherage DE, Blanchard JL (2016). Tempo and mode of genome evolution in a 50,000-generation experiment. Nature.

[52] Stevenson G, Andrianopoulos K, Hobbs M, Reeves PR (1996). Organization of the *Escherichia coli* K-12 gene cluster responsible for production of the extracellular polysaccharide colanic acid. J Bacteriol.

[53] Schneider D, Duperchy E, Depeyrot J, Coursange E, Lenski R (2002). Genomic comparisons among *Escherichia coli* strains B, K-12, and O157:H7 using IS elements as molecular markers. BMC Microbiol.

[54] Good BH, McDonald MJ, Barrick JE, Lenski RE, Desai MM (2017). The dynamics of molecular evolution over 60,000 generations. Nature.

[55] Lang GI, Rice DP, Hickman MJ, Sodergren E, Weinstock GM (2013). Pervasive genetic hitchhiking and clonal interference in forty evolving yeast populations. Nature.

[56] Maddamsetti R, Lenski RE, Barrick JE (2015). Adaptation, clonal interference, and frequency-dependent interactions in a long-term evolution experiment with *Escherichia coli*. Genetics.

[57] Bailey SF, Blanquart F, Bataillon T, Kassen R (2017). What drives parallel evolution? How population size and mutational variation contribute to repeated evolution. Bioessays.

[58] Buskirk SW, Peace RE, Lang GI (2017). Hitchhiking and epistasis give rise to cohort dynamics in adapting populations. Proc Natl Acad Sci USA.

[59] Brody H, Greener A, Hill CW (1985). Excision and reintegration of the *Escherichia coli* K-12 chromosomal element e14. J Bacteriol.

[60] Borin JM, Avrani S, Barrick JE, Petrie KL, Meyer JR (2021). Coevolutionary phage training leads to greater bacterial suppression and delays the evolution of phage resistance. Proc Natl Acad Sci USA.

[61] Uz-Zaman MH, D’Alton S, Barrick JE, Ochman H (2024). Promoter recruitment drives the emergence of proto-genes in a long-term evolution experiment with *Escherichia coli*. PLoS Biol.

[62] Katoh K, Standley DM (2013). MAFFT multiple sequence alignment software version 7: improvements in performance and usability. Mol Biol Evol.

