# Supplementary material for: Parallel evolution of chimeric genes in microbial evolution experiments
Source: Microb Genom. 2026 Sep 30;12(9):001850. doi: 10.1099/mgen.0.001850 (PMC13626634; doi:10.1099/mgen.0.001850)
Supplement: Supplementary file 1 [file mgen-12-01850-s001.pdf]

SUPPLEMENTARY TABLES AND FIGURES

Table S1. Gene amplifications in experiment reported by Mohiuddin et al. (2025).

Population	Gene	K-12 locus tag	Amplification start	Length	Mean copy number	Bonferroni-corrected p-value
1	*bamA*	b0177	198903	1144	2.42	0.0016
2	*bamA*	b0177	199111	1275	2.80	4.61E-05
3	*bamA*	b0177	199368	903	2.67	0.0062
3	*lldP*	b3603	3778655	1099	2.71	0.00070
4	*bamA*	b0177	199079	1130	2.42	0.00024
4	*lldP*	b3603	3778674	933	2.64	0.030
4	*rpoB*	b3987	4183283	1194	2.34	0.00074
7	*bamA*	b0177	199079	1318	2.53	9.29E-07
7	*rpoB*	b3987	4183288	1005	2.17	0.035
8	*bamA*	b0177	199079	1320	2.40	1.03E-05
